# Supplementary material for: The molecular basis underlying T cell specificity towards citrullinated epitopes presented by HLA-DR4
Source: Nat Commun. 2024 Jul 23;15:6201. doi: 10.1038/s41467-024-50511-w (PMC11266596; doi:10.1038/s41467-024-50511-w)
Supplement: Supplementary file 1 — Supplementary Information [file 41467_2024_50511_MOESM1_ESM.pdf]

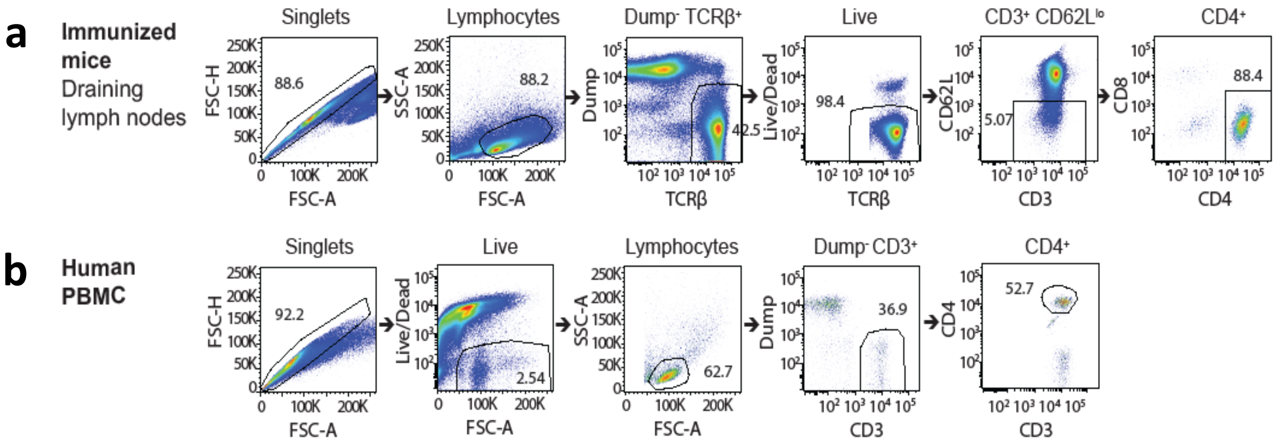

Supplementary Fig 1. Gating strategies for sorting HLA-DR4$^{\text{Vim-64cit59-71}}$- and HLA-DR4$^{\alpha\text{-eno-15cit10-22}}$-specific CD4 T cells. **a** Representative gating strategy for the detection of HLA-DR4$^{\text{Vim-64cit59-71}}$- and HLA-DR4$^{\alpha\text{-eno-15cit10-22}}$-specific CD4$^+$ T cells from the draining lymph nodes of immunized HLA-DR4 mice, which includes gating on CD62L^{lo}CD3$^+$ cells prior to gating on CD4$^+$ cells. Numbers in black type indicate percentage of cells within a gate. **b** Representative gating strategy for the detection of HLA-DR4$^{\text{Vim-64cit59-71}}$, HLA-DR4$^{\alpha\text{-eno-15cit10-22}}$ and HLA-DR4$^{\alpha\text{-eno-15cit10-22V20G}}$- specific CD4$^+$ T cells in PBMC isolated from HLA-DR4$^+$ RA donors or healthy controls. Numbers in black type indicate percentage of cells within a gate. Shown is sample from RA donor 2.

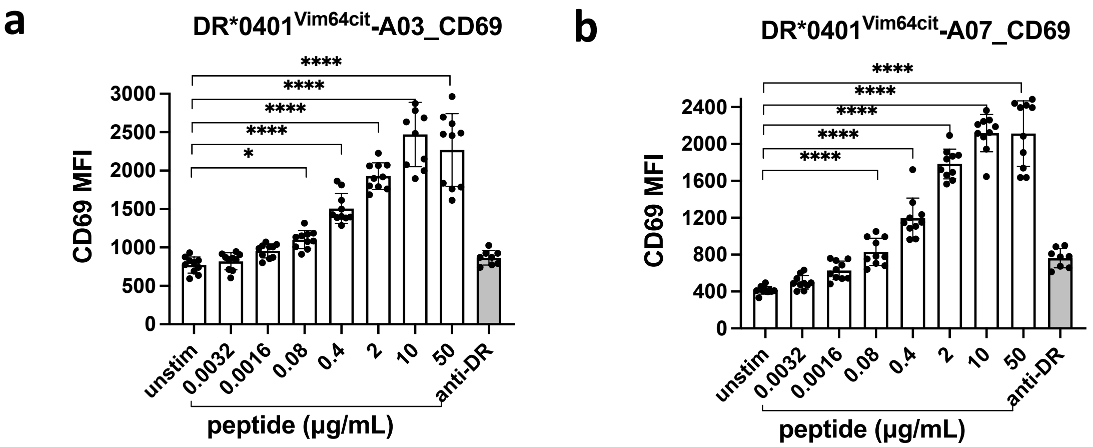

Supplementary Fig. 2. Antigen reactivity of A03 and A07 TCRs toward HLA-DR4$^{\mathrm{Vim\text{-}64cit59\text{-}71}}$. Expression of CD69 on the surface of **a** A03 TCR transduced SKW-3 cell lines or **b** A07 TCR transduced SKW-3 cell lines stimulated overnight with serial dilution of vim64-cit 59-71 peptide-pulsed BLCL 9031. All samples were performed in duplicates and the black dots are presented as the mean fluorescence intensity (MFI) of average of duplicated values from five independent experiments (n=5). Anti-HLA-DR4 antibody used as control. P values were determined by one way ANOVA with Dunnett's multiple comparison testing, *$P < 0.05$, **$P < 0.01$, ***$P < 0.002$, ****$P < 0.0001$ and error bars represent ± s.e.m.. Source data are provided as a Source Data file.

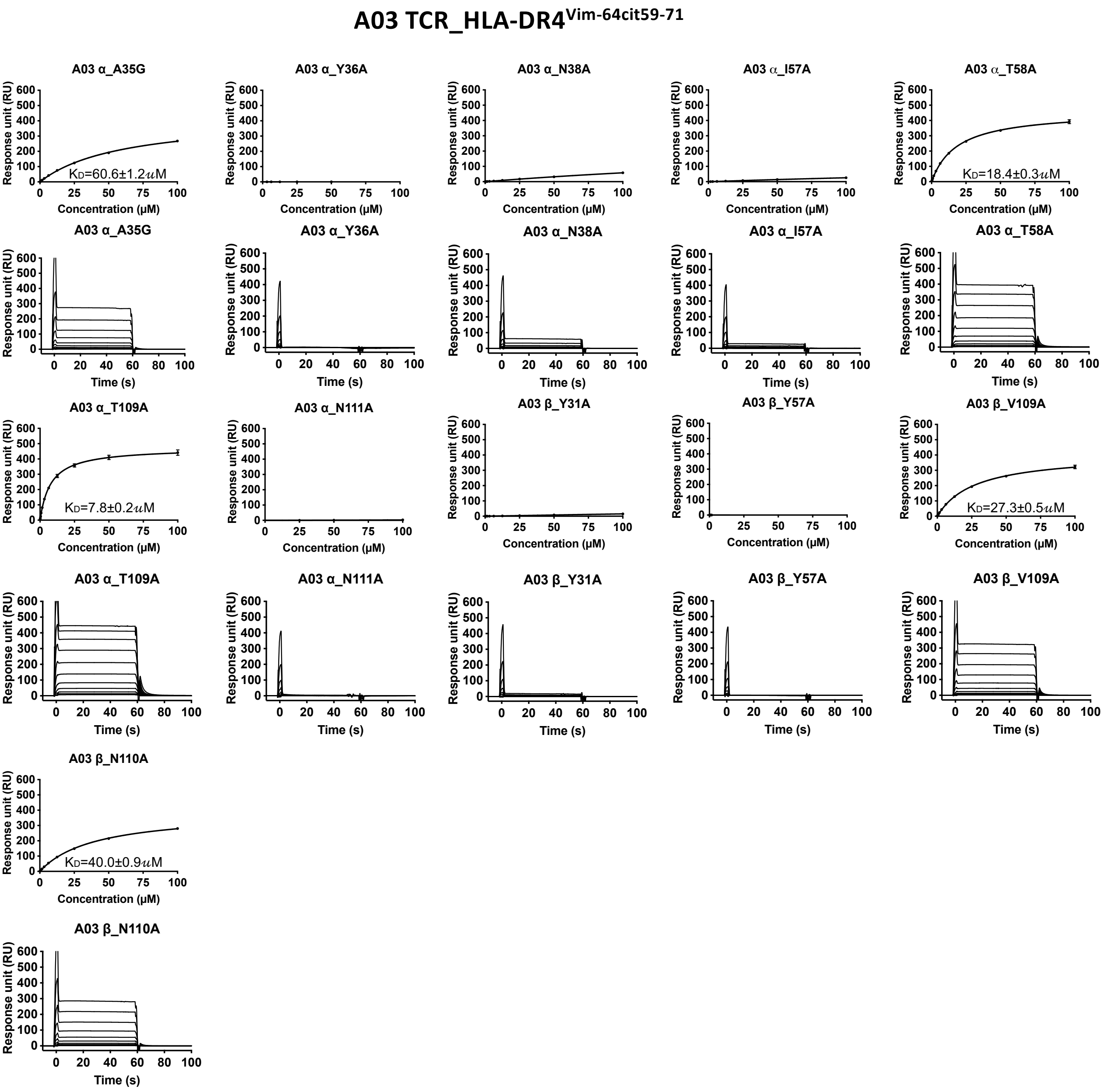

Supplementary Fig. 3. Affinity analysis of point mutations on A03 TCR toward HLA-DR4$^{\text{Vim-64cit59-71}}$. The equilibrium affinity constants (K_D) of A03 TCR toward HLA-DR4$^{\text{Vim-64cit59-71}}$ for point mutations was determined by SPR. All data derived from three independent experiments (n=3) and a single ligand binding model was used for curve fitting. The maximal TCRs concentration used is 100 μM equal to wild type TCR concentration. HLA-DR4^{CLIP} was used as reference flow cell to control nonspecific binding. Equilibrium response curves were normalised against the calculated maximum response and the measurements then combined. Data are mean ± standard error, s.e.m.. Source data are provided as a Source Data file.

A03 TCR_HLA-DR4$^{Vim\text{-}64cit59\text{-}71}$ mutants

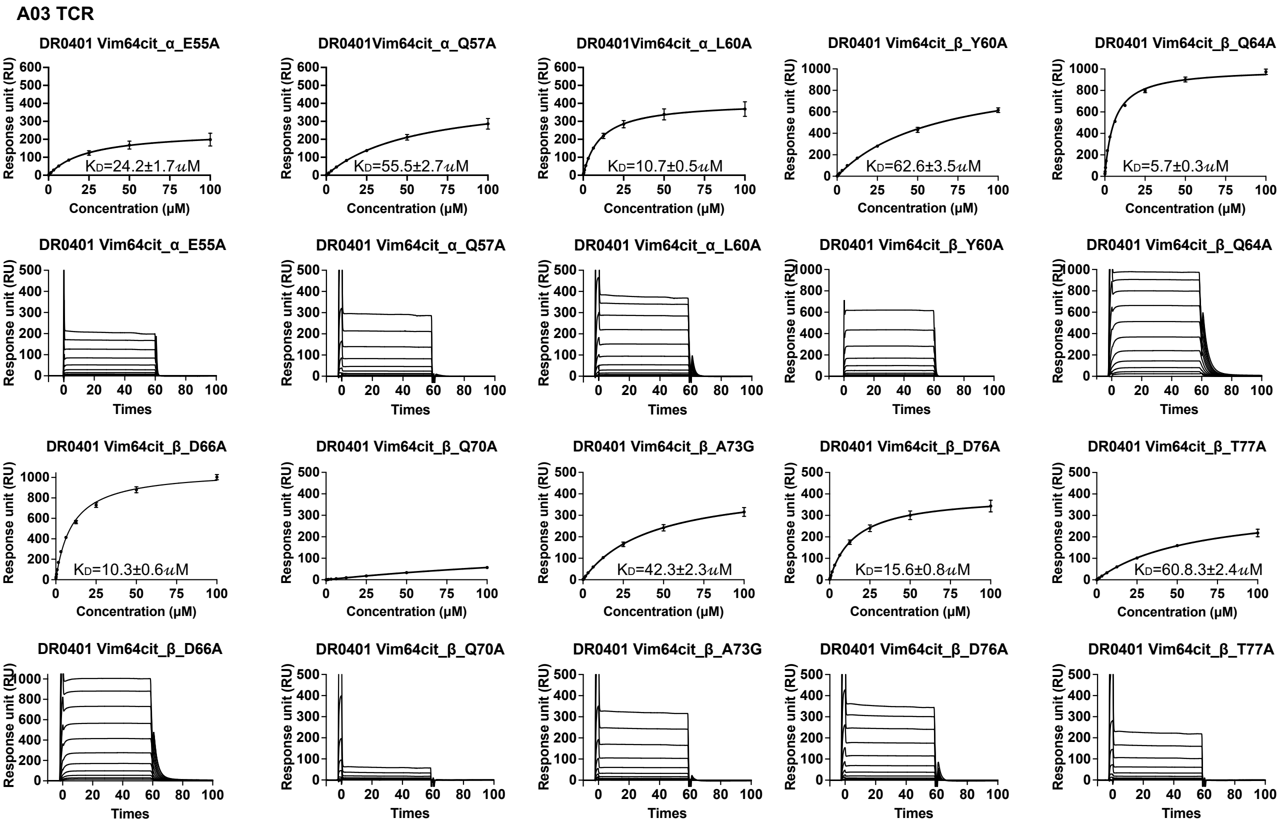

Supplementary Fig. 4. Affinity analysis of A03 TCR toward point mutation of HLA-DR4$^{Vim\text{-}64cit59\text{-}71}$. Binding analysis of A03 TCR toward each point mutation of HLA-DR4$^{Vim\text{-}64cit59\text{-}71}$ was determined by SPR. All data derived from two independent experiments (n=2) and K_D determination using a single ligand binding model. To control nonspecific binding, HLA-DR4^{CLIP} was used as reference flow cell. Equilibrium response curves were normalised against the calculated maximum response and the measurements then combined. Data are mean ± standard error, s.e.m.. Source data are provided as a Source Data file.

RA2.7 TCR_ HLA-DR4$^{\alpha\text{-eno-15cit10-22V20G}}$

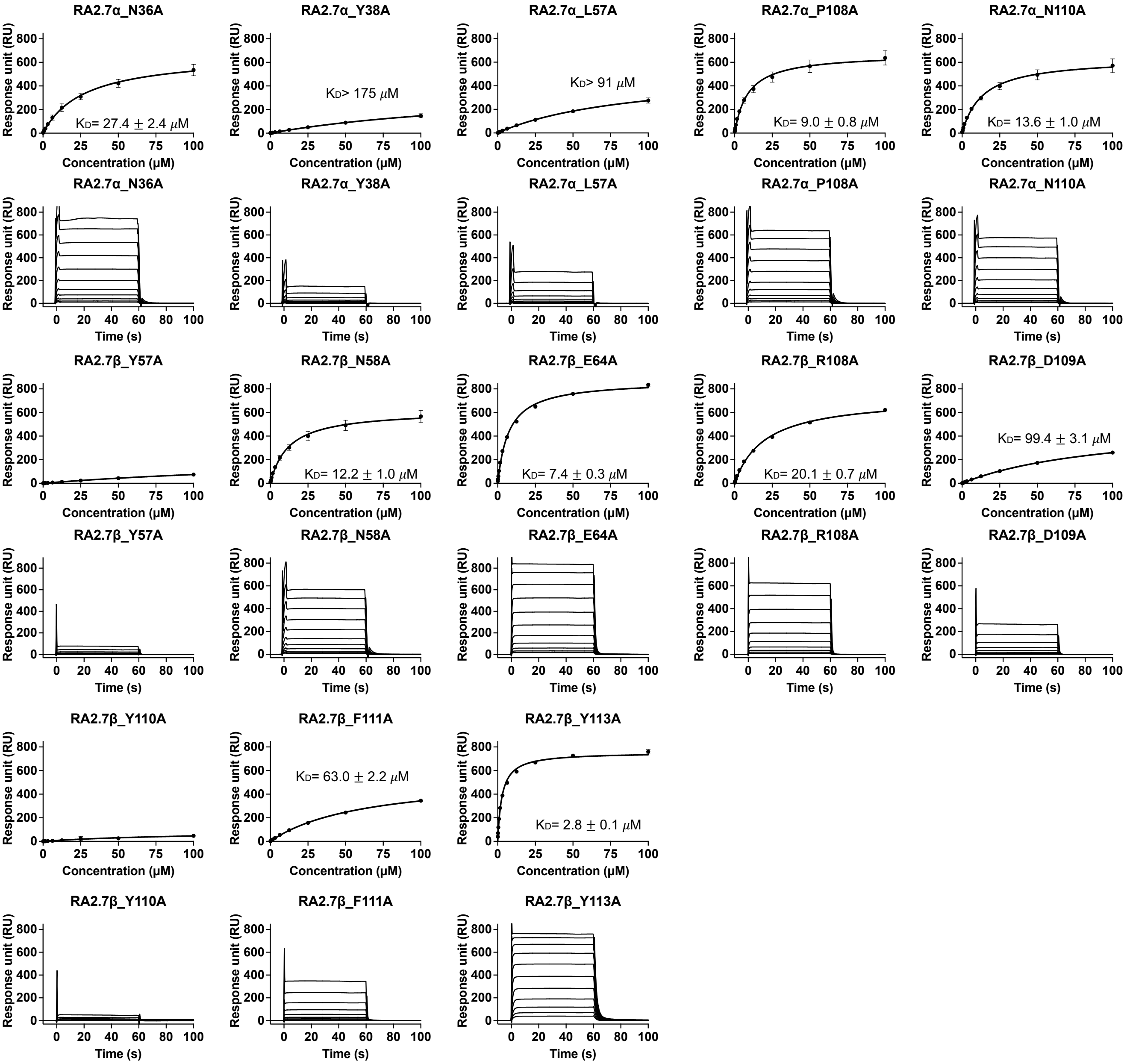

Supplementary Fig. 5. Binding affinity measurement of RA2.7 TCR point mutations towards HLA-DR4$^{\alpha\text{-eno-15cit10-22V20G}}$. All SPR data were derived from three independent experiments (n=3) for K_D determination and curve fitting with a single ligand binding model. Maximal TCRs concentration used is 100 μM and HLA-DR4^{CLIP} was used as reference flow cell to control nonspecific binding. Equilibrium response curves were normalised against the calculated maximum response and the measurements then combined. Data are mean ± standard error, s.e.m.. Source data are provided as a Source Data file.

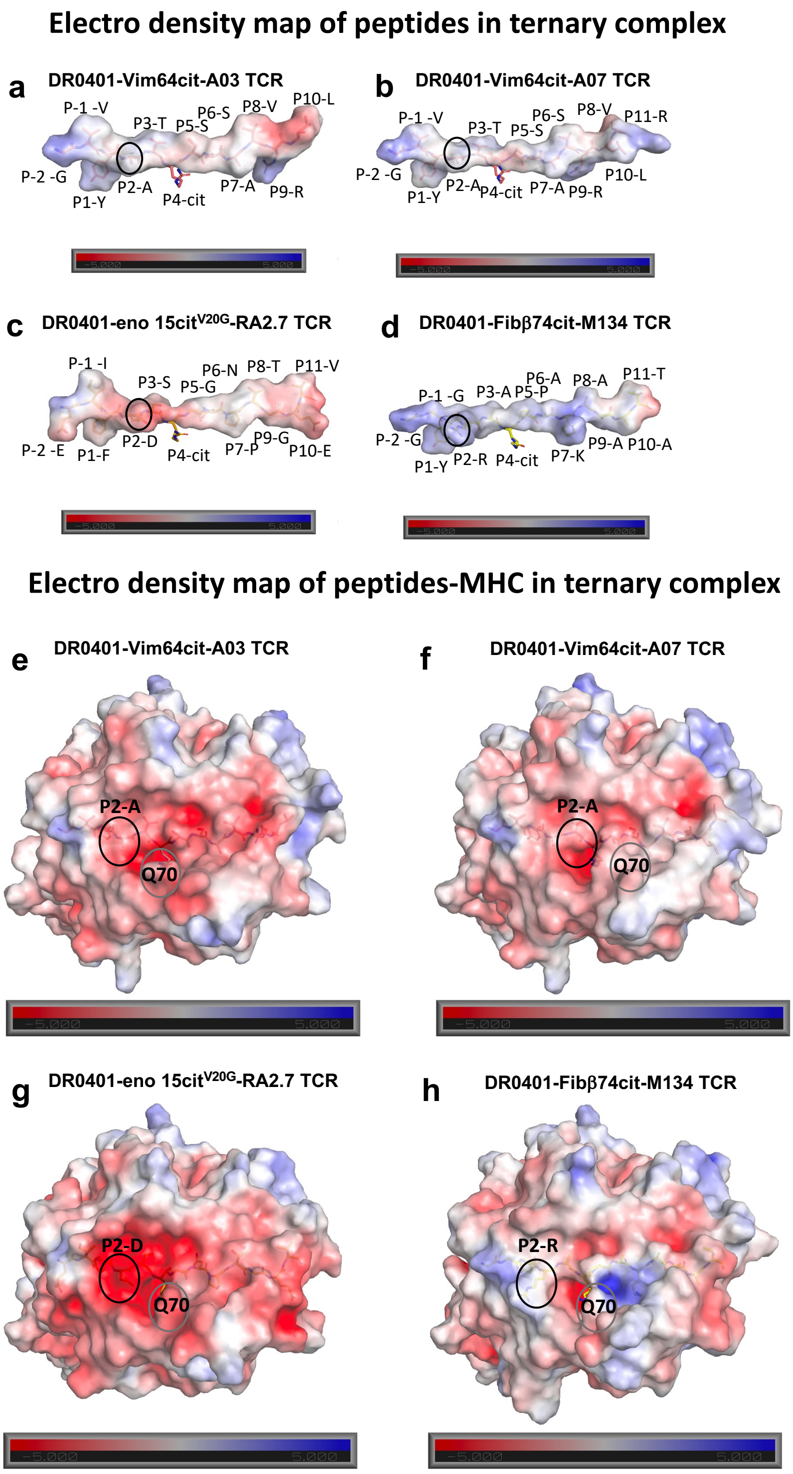

Supplementary Fig. 6. Adaptive Poisson Boltzmann Solver-generated electrostatic surface of peptide or pHLA in ternary complexes (red, negative; blue, positive; white, neutral). The view from the side, looking straight at the peptide binding groove of peptide **a** HLA-DR4$^{Vim\text{-}64cit59\text{-}71}$-A03 TCR complex, **b** HLA-DR4$^{Vim\text{-}64cit59\text{-}71}$-A07 TCR complex, **c** HLA-DR4$^{\alpha\text{-}eno\text{-}15cit10\text{-}22V20G}$-RA2.7 TCR complex, **d** HLA-DR4-$^{Fib\beta\text{-}74cit}$-M134 TCR complex. The view from the top, looking straight into the peptide binding groove from **e** HLA-DR4$^{Vim\text{-}64cit59\text{-}71}$-A03 TCR complex, **f** HLA-DR4$^{Vim\text{-}64cit59\text{-}71}$-A07 TCR complex, **g** HLA-DR4$^{\alpha\text{-}eno\text{-}15cit10\text{-}22V20G}$-RA2.7 TCR complex, **h** HLA-DR4-$^{Fib\beta\text{-}74cit}$-M134 TCR complex. Black circle is the position where P2 residue is located, grey circle is the position where the SE Gln^{70}β is located on the HLA-DR4 β-chain.

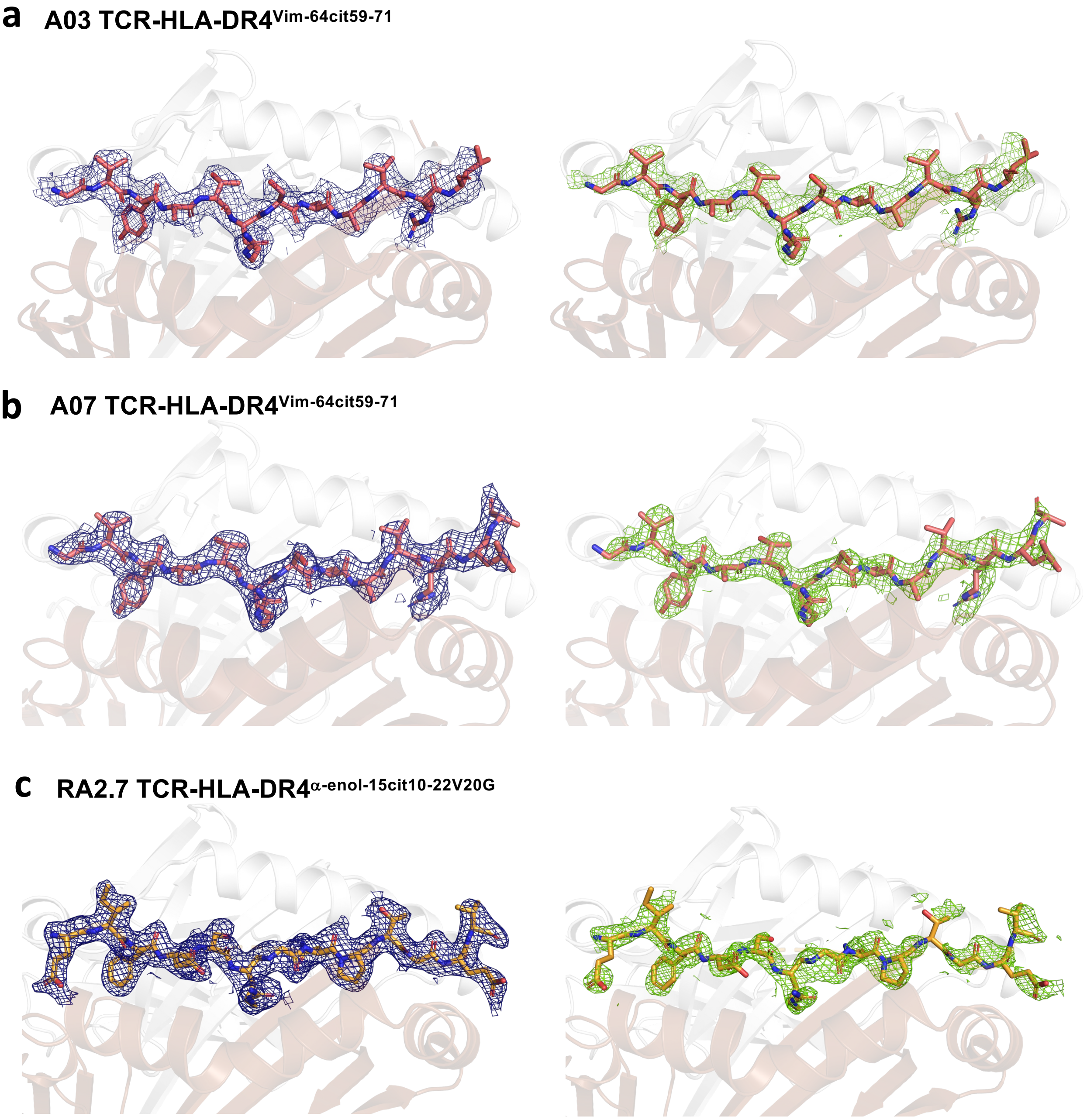

Supplementary Fig. 7. Peptide antigen electron density map. Electron density map of the peptide antigen for **a** A03 TCR-HLA-DR4$^{Vim\text{-}64cit59\text{-}71}$, **b** A07 TCR-HLA-DR4$^{Vim\text{-}64cit59\text{-}71}$, and **c** RA2.7 TCR-HLA-DR4$^{\alpha\text{-}enol\text{-}15cit10\text{-}22V20G}$ complexes are presented in refined 2mFo-DFc map (left; blue mesh) and SA Omit map (right; green mesh), respectively, contoured at 1σ. The HLA-DR4 α and β chains are coloured in white and brown, respectively. Vim-64cit$_{59\text{-}71}$ and α-eno15cit$_{10\text{-}22}$ peptides are coloured in pink and orange, respectively.

Supplementary Table 1. Paired TCRαβ sequences of confirmed HLA-DR4$^{\alpha\text{-eno-15cit10-22V20G}}$-specific $CD4^+$ cells isolated from peripheral blood of $ACPA^+$ RA donor and HLA-$DR4^+$ healthy control donors. Epitope specificity was verified by tetramer staining of TCR transfectants.

**Subject**	**TRAV**	**TRAJ**	**CDR1α**	**CDR2α**	**CDR3α**	**TRBV**	**TBRD**	**TRBJ**	**CDR1β**	**CDR2β**	**CDR3β**
**RA donor 2**	26-1	49	TISGNEY	GLKNN	CIVRASNTGNQFYF	20-1	2	2-7	DFQATT	SNEGSKA	CSAATGTSGYEQYF
**RA donor 2**	27	26	SVFSS	VVTGGEV	CAGKAGNYGQNFVF	19	1	2-2	LNHDA	SQIVND	CASSTGGQNTGELFF
**HC 10195**	26-1	53	TISGNEY	GLKNN	CIVRTSGGSNYKLTF	2	1	2-1	SNHLY	FYNNEI	CAGTYWNEQFF
**HC 4737**	26-1*	56	TISGNEY	GLKNN	CIVRVAGANSKLTF	5-4	1	2-7	SGHNT	YYREEE	CASSYDLAGPYEQYF
**HC 4737**	35	39	SIFNT	LYKAGEL	CAGRNNAGNMLTF	2	2	1-2	SNHLY	FYNNEI	CASNVGIINYGYTF

RA = rheumatoid arthritis, HC = healthy control.

* Two cells isolated for this clone

CDR3 nucleotide sequences are listed in Supplementary Table 10.

Supplementary Table 2. Data collection and refinement statistics

	A03 TCR-HLA-DR4$^{Vim-64cit59-71}$	A07 TCR-HLA-DR4$^{Vim-64cit59-71}$	RA2.7 TCR-HLA-DR4$^{\alpha-enolase-15cit10-22V20G}$
**Data collection**			
Space group	I121	P 2 21 21	C121
Cell dimensions			
a, b, c (Å)	98.93, 59.97, 379.16	58.60, 76.62, 224.18	185.86, 58.55, 216.48
a, b, g (°)	90, 90.16, 90	90, 90, 90	90, 113.60, 90
Resolution (Å)	49.52-2.65 (2.72-2.65)	46.55-2.75 (2.90-2.75)	45.87-2.40 (2.44-2.40)
R_{sym} or R_{merge}	0.102 (1.135)	0.161 (1.825)	0.07 (0.88)
I / sI	11.9 (1.9)	12.4 (1.7)	12.5 (2.1)
Completeness (%)	100 (100)	100 (100)	100.0 (100.0)
Redundancy	7.0 (7.2)	13.4 (13.8)	6.2 (6.1)
**Refinement**			
Resolution (Å)	49.486-2.65	46.548-2.75	45.87-2.40
No. reflections	65333	27098	84262
R_{work} / R_{free}	0.2086/0.2441	0.2160/0.2635	0.2053/0.2322
No. atoms	13047	6638	12917
Protein	12704	6513	12325
Ligand/ion	252	88	249
Water	91	37	343
B-factors	79.48	71.21	62.89
Protein	79.47	71.17	63.34
Ligand/ion	103.80	89.38	70.82
Water	67.79	66.51	52.44
R.m.s. deviations			
Bond lengths (Å)	0.002	0.003	0.003
Bond angles (°)	0.539	0.521	0.606
Rama allowed (%)	99.81	99.88	99.94
Rama favoured (%)	96.89	95.22	96.5
Rama Outlier (%)	0.19	0.12	0.06

Supplementary Table 3. TCR-pHLA complex statistics.

**TCR-pHLA**	**BSA (Å²)**	**Vα (%)**	**Vβ (%)**	**Peptide (%)**	**1α (%)**	**2α (%)**	**3α (%)**	**Fα (%)**	**1β (%)**	**2β (%)**	**3β (%)**	**Fβ (%)**	**K$_D$ (μM)**	**Angle (°)**
A03-HLA-DR4$^{Vim6\text{-}4cit59\text{-}71}$	2010	54.5	45.5	27.1	20.7	9.6	20.8	3.4	6.3	10.7	27.5	1	6.2± 0.3μM	67
A07-HLA-DR4$^{Vim\text{-}64cit59\text{-}71}$	1665	63.7	36.3	28.7	23.1	8.7	29.6	2.3	0	0	36.3	0	31.9± 1.5μM	61
RA2.7-HLA-DR4$^{Vim\text{-}64cit59\text{-}71v20G}$	1940	38	62	20.95	17	3	17	1	7	17	33	5	7.1± 0.5μM	60

Supplementary Table 4. Contact table of A03 TCR- HLA-DR4$^{Vim\text{-}64cit59\text{-}71}$

TCR segment	TCR residues	HLA-DR4	Type of bond
**CDR1α**	A35	H81β	HB, VDW
	Y36	D76β, T77β	VDW
	N38	Q70β	HB, VDW
**FWα**	K55	D66β	SB
**CDR2α**	I57	Q70β	VDW
	T58	A73β, T77β	VDW
**CDR3α**	T109	Q70β	HB, VDW
	G110	G58α	VDW
	N111	Q57α, G58α	VDW
	Y112	Q57α	HB, VDW
	K113	E55α	HB, VDW
**CDR1β**	Y31	Q57α, A61α	HB, VDW
**FWβ**	Y55	Q57α	VDW
**CDR2β**	Y57	Q57α, L60α	VDW
**CDR3β**	N110	Y60β, Q64β, L67β	HB, VDW
	S111	D66β	VDW
**TCR segment**	**TCR residues**	**Vim-64cit$_{59\text{-}71}$ peptide**	**Type of bond**
**CDR1α**	T28	P-1-V	VDW
	S29	P-1-V	VDW
	I30	P-1-V	VDW
	A31	P-1-V, P1-Y, P2-A	VDW
**CDR3α**	T109	P4-Cit, P5-S	VDW, HB
	G110	P3-T, P5-S	VDW
**CDR1β**	D30	P8-V	VDW
**CDR3β**	V109	P5-S, P6-S	VDW
	N110	P8-V	HB, VDW

VDW: Van der Waals interaction (cut-off at 4Å)
HB: hydrogen bond (cut-off at 3.5 Å)
SB: salt bridge (cut-off at 4.5 Å)

Supplementary Table 5. Contact table of A07 TCR- HLA-DR4$^{Vim\text{-}64cit59\text{-}71}$

TCR segment	TCR residues	HLA-DR4	Type of bond
**CDR1α**	S29	E55α	VDW
	A35	H81β	VDW
	Y36	T77β, H81β	VDW
**FWα**	K55	D66β	SB
**CDR2α**	I57	E69β, T77β	VDW
	T58	A73β	VDW
**CDR3α**	H109	Q70β, A73β, T77β	HB, VDW
	S110	Q70β	HB, VDW
	S112	A61α	VDW
	W113	E55α	VDW
**CDR3β**	R109	V65α	VDW
	T110	Q64β, L67β	VDW
	G111	Q70β	HB, VDW
	A112	D66β, L67β, Q70β	VDW
	D115	D66β	VDW
**TCR segment**	**TCR residues**	**Vim-64cit$_{59\text{-}71}$ peptide**	**Type of bond**
**CDR1α**	A31	P-1V, P1-Y, P2-A	VDW
**CDR3α**	H109	P4-Cit	HB, VDW
	S110	P3-T, P4-Cit, P5-S	VDW
**CDR3β**	R109	P8-V	VDW
	T110	P7-A, P8-V	VDW
	G111	P5-S, P8-V	HB, VDW

VDW: Van der Waals interaction (cut-off at 4Å)
HB: hydrogen bond (cut-off at 3.5 Å)
SB: salt bridge (cut-off at 4.5 Å)

Supplementary Table 6. Contact table of RA2.7 TCR- HLA-DR4$^{\alpha\text{-enolase-15cit10-22V20G}}$

TCR segment	TCR residues	HLA-DR4	Type of bond
**CDR1α**	G30	T77β, H81β	VDW
	N36	T77β, H81β	VDW, HB
	Y38	Q70β, A73β	VDW
**CDR2α**	L57	T77β	VDW
**CDR3α**	A109	Q70β	VDW
	N110	G58a, N62a	VDW, HB
**CDR1β**	Y38	V65a	VDW
**CDR2β**	Y57	A64a, V65a, A68a	VDW
	N58	A68a	VDW
	E64	K67a	SB
**FWβ**	S66	Q57a	HB, VDW
	E67	Q57a	VDW
**CDR3β**	R108	Y60β, Q64β	HB
	Y110	Q70β, K71β	HB, VDW
	F111	Q64β, L67β, Q70β	VDW
	Y113	D66β	VDW, HB
**TCR segment**	**TCR residues**	**α-enolase-15cit$_{10\text{-}22V20G}$ peptide**	**Type of bond**
**CDR1α**	N36	P2-D	HB, VDW
**CDR3α**	P108	P2-D	VDW
	N110	P3-S, P4-Cit, P5-G	HB, VDW
**CDR1β**	L37	P8-T	VDW
**CDR2β**	Y57	P8-T	HB, VDW
**CDR3β**	D109	P7-P, P8-T	HB, VDW
	Y110	P4-cit, P5-G, P6-N, P7-P	HB, VDW
	F111	P7-P	VDW

VDW: Van der Waals interaction (cut-off at 4 Å)
HB: hydrogen bond (cut-off at 3.5 Å)
SB: salt bridge (cut-off at 4.5 Å)

Supplementary Table 7. Antibody list

Antibody name	Clone	Company	Dilution used	Catalog number
BUV395 mouse anti-human CD3	UCHT1	BD Biosciences	1:100	563546
APC Mouse Anti-Human CD69	FN50	BD Biosciences	1:100	555533
anti-HLA-DR4	LB3.1			
APC-Cy™7 Hamster Anti-Mouse TCR β Chain	H57-597	BD Biosciences	1:200	560656
FITC Rat Anti-Mouse CD45R/B220	RA3-6B2	BD Biosciences	1:400	553088
FITC Mouse Anti-Mouse NK-1.1	PK136	BD Biosciences	1:400	553164
FITC Rat anti-Mouse F4/80	BM8	eBioscience, ThermoFisher Scientific	1:400	11-4801-82
BV711 Hamster Anti-Mouse CD3e	145-2C11	BD Biosciences	1:200	563123
BV605 Rat Anti-Mouse CD62L	MEL-14	BD Biosciences	1:400	563252
BUV395 Rat Anti-Mouse CD4	GK1.5	BD Biosciences	1:200	563790
Pacific Blue™ Rat Anti-Mouse CD8a	53-6.7	BD Biosciences	1:200	558106
Alexa Fluor 700 Mouse anti-human CD14	M5E2	BD Biosciences	1:100	557923
Alexa Fluor 700 Mouse anti-human CD19	HIB19	BD Biosciences	1:200	557921
BV480 Mouse anti-human CD3	UCHT1	BD Biosciences	1:200	566105
BUV395 mouse anti-human CD4	SK3	BD Biosciences	1:400	563550
APC hamster anti-mouse TCR β chain	H57-597	Biolegend	1:400	109212
APC mouse anti-human CD3	UCHT1	Biolegend	1:200	300412
LIVE/DEAD™ Fixable Aqua Dead Cell Stain Kit, for 405 nm excitation		ThermoFisher Scientific	1:800	L34966
LIVE/DEAD Fixable Near-IR Dead Cell Stain Kit, for 633 or 635 nm excitation		ThermoFisher Scientific	1:1000	L10119
Zombie NIR™ Fixable Viability Kit		Biolegend	1:1000	423106
BD Horizon™ Fixable Viability Stain 700 (FVS700)		BD Biosciences	1:1000	564997

Supplementary Table 8. Primers targeting mouse T cell receptor α (TRA) and β (TRB) genes

TRA gene(s) targeted by primer	External primer sequence	Internal primer sequence	Reference
TRAV1	5’ GCACATACAGCACCTCAG 3’	5’ AACGTGAAGGCCAAGC 3’	
TRAV2	5’ CCACCAGGGACCACAG 3’	5’ ACTCTGAGCCTGCCCT 3’	50 (Internal only)
TRAV3	5’ GGCGAGCAGGTGGAG 3’	5’ GCCCTCCTCACCTGAG 3’	50
TRAV4	5’ TCTGSTCTGAGATGCAATTTT 3’	5’ GGITIMAGGAACAAAGGAGAAT 3’	50
TRAV5-1/5-4(D)	5’ CTTCCYTTGGTATAAGCAAGA 3’	5’ ATYCGTTCAAATATGGAAAGAAA 3’	50 (Internal only)
TRAV6-1/6-2	5’ CAGATGCAAGGTCAAGTGAC 3’	5’ GGAGAAGGTCCACAGCTC 3’	50
TRAV6-3/6-4(D)	5’ AAGGTCCACAGCTCCTTC 3’	5’ CAACTGCCAACAACAAGG 3’	50
TRAV6(D)-5	5’ CTTCTCTGACTGTGAACTGTTC 3’	5’ CAGTACCCAACCCTGTTCTG 3’	
TRAV6-6	5’ AGATTCCGTGACTCAAACAG 3’	5’ ACGGCTGGCCAGAAG 3’	50
TRAV6(D/N)-7	5’ GCCTCAAGGGACAAAGAG 3’	5’ AAAGGAAGCAGCAGAGG 3’	
TRAV7	5’ AGAAGGTRCAGCAGAGCCCAGAATC 3’	5’ CAKGRCYTCYYTCAACTGCAC 3’	50
TRAV8	5’ TGAAYTGYAGTTACAAGAC 3’	5’ TAATCTTAATACGTTCAAATGAG 3’	
TRAV9	5’ CTCKSTGSAGCTGAGATGCAA 3’	5’ CAGYTKCTCCTCAAGTACTAT 3’	
TRAV10	5’ GGAGAGAAGGTCGAGCAAC 3’	5’ GAGGGAGACAGCGCTG 3’	
TRAV11	5’ AAGACCCAAGTGGAGCAG 3’	5’ AACAGGACACAGGCAAAG 3’	50
TRAV12	5’ GACCCAGAMRGAAGGCCTG 3’	5’ GCTGAACWGCACCTATCAG 3’	
TRAV12-4	5’ GGGAGGAGCAATGGAGATGG 3’	5’ CAGTGACCCAGAAGGAAGG 3’	
TRAV13	5’ TCCTTGGTTCTGCAGG 3’	5’ TGCAGTGGTTTTACCAA 3’	50 (External only)
TRAV14	5’ GCAGCAGGTGAGACAAAG 3’	5’ CTCTGACAGTCTGGGAAGG 3’	50
TRAV15	5’ CTGSAYTGTTCATATRAGACAAGT 3’	5’ TTAGTGGAGAGATGGTTTT 3’	
TRAV16	5’ GTACAAGCAAACAGCAAGTG 3’	5’ ATTATTCTCTGAACTTTCAGAAGC 3’	50
TRAV17	5’ CAGTCCGTGGACCAGC 3’	5’ TATGAAGGAGCCTCCCTG 3’	50
TRAV18	5’ CAAGATTTCACTGCACG 3’	5’ TACTGGTACCGACAGGTC 3’	
TRAV19	5’ CAAGTTAAACAAAGCTCTCCATC 3’	5’ GCTGACTGTTCAAGAGGGA 3’	50 (Internal only)
TRAV21	5’ GTGCACTTGCCTTGTAGC 3’	5’ AATAGTATGGCTTTCCTGGC 3’	50
TRAC	5’ GGCATCACAGGGAACG 3’	5’ GCACATTGATTTGGGAGTC 3’	50

TRB gene(s) targeted by primer	External primer sequence	Internal primer sequence	Reference
TRBV1	5’ TACCACGTGGTCAAGCTG 3’	5’ GTATCCCTGGATGAGCTG 3’	50
TRBV2	5’ CAGTATCTAGGCCACAATGC 3’	5’ GGACAATCAGACTGCCTC 3’	50
TRBV3	5’ CCCAAAGTCTTACAGATCCC 3’	5’ GATATGGGGCAGATGGTG 3’	50
TRBV4	5’ GACGGCTGTTTTCCAGAC 3’	5’ CAGGTGGGAAATGAAGTG 3’	50
TRBV5	5’ GGTATAAACAGAGCGCTGAG 3’	5’ GCCAGAGCTCATGTTTCTC 3’	50
TRBV12	5’ GGGGTTGTCCAGTCTCC 3’	5’ CCAGCAGATTCTCAGTCC 3’	50
TRBV13	5’ GCTGCAGTCACCCAAAG 3’	5’ GTACTGGTATCGGCAGGAC 3’	50
TRBV14	5’ GCAGTCCTACAGGAAGGG 3’	5’ GGTATCAGCAGCCCAGAG 3’	50
TRBV15	5’ GAGTTACCCAGACACCCAG 3’	5’ GTGTGAGCCAGTTTCAGG 3’	50
TRBV16	5’ CCTAGGCACAAGGTGACAG 3’	5’ GAAGCAACTCTGTGGTGTG 3’	50
TRBV17	5’ GAAGCCAAACCAAGCAC 3’	5’ GAACAGGGAAGCTGACAC 3’	50
TRBV19	5’ GATTGGTCAGGAAGGGC 3’	5’ GGTACCGACAGGATTCAG 3’	50
TRBV20	5’ GGATGGAGTGTCAAGCTG 3’	5’ GCTTGGTATCGTCAATCG 3’	50
TRBV23	5’ CTGCAGTTACACAGAAGCC 3’	5’ GCCAGGAAGCAGAGATG 3’	50
TRBV24	5’ CAGACTCCACGATACCTGG 3’	5’ GCACACTGCCTTTTACTGG 3’	50
TRBV26	5’ GGTGAAAGGGCAAGGAC 3’	5’ GAGGTGTATCCCTGAAAAGG 3’	50
TRBV29	5’ GCTGGAATGTGGACAGG 3’	5’ GTACTGGTATCGACAAGACCC 3’	50
TRBV30	5’ CCTCCTCTACCAAAAGCC 3’	5’ GGACATCTGTCAAAGTGGC 3’	50
TRBV31	5’ CTAACCTCTACTGGTACTGGCAG 3’	5’ CTGTTGGCCAGGTAGAGTC 3’	50
TRBC	5’ CCAGAAGGTAGCAGAGACCC 3’	5’ CCTCCTTGCCATTCACCCAC 3’	50 (External only)

Primers targeting TRAV and TRBV genes are sense. Primers targeting TRAC and TRBC genes are antisense. TRAV, T cell receptor Vα, TRAC, T cell receptor Cα; TRBV, T cell receptor Vβ, TRBC, T cell receptor Cβ.

Supplementary Table 9. Primers targeting human T cell receptor α (TRA) and β (TRB) genes

TRA gene(s) targeted by primer	External primer sequence	Internal primer sequence	Reference
TRAV1	5’ AACTGCACGTACCAGACATC 3’	5’ GCACCCACATTTCTKTCTTAC 3’	51
TRAV2	5’ GATGTGCACCAAGACTCC 3’	5’ CACTCTGTGTCCAATGCTTAC 3’	51
TRAV3	5’ AAGATCAGGTCAACGTTGC 3’	5’ ATGCACCTATTCAGTCTCTGG 3’	51
TRAV4	5’ CTCCATGGACTCATATGAAGG 3’	5’ ATTATATCACGTGGTACCAACAG 3’	51
TRAV5	5’ CTTTTCCTGAGTGTCCGAG 3’	5’ TACACAGACAGCTCCTCCAC 3’	51
TRAV6	5’ CACCCTGACCTGCAACTATAC 3’	5’ TGGTACCGACAAGATCCAG 3’	51
TRAV7	5’ GCAAAATACAGGGATGGG 3’	5’ TATGAGAAGCAGAAAGGAAGAC 3’	
TRAV8-1	5’ CTCACTGGAGTTGGGATG 3’	5’ GTCAACACCTTCAGCTTCTC 3’	51
TRAV8-2, 8-4	5’ GCCACCCTGGTTAAAGG 3’	5’ AGAGTGAAACCTCCTTCCAC 3’	51
TRAV8-3	5’ CACTGTCTCTGAAGGAGCC 3’	5’ TTTGAGGCTGAATTTAAGAGG 3’	51
TRAV8-6	5’ GAGCTGAGGTGCAACTACTC 3’	5’ AACCAAGGACTCCAGCTTC 3’	51
TRAV8-7	5’ CTAACAGAGGCCACCCAG 3’	5’ ATCAGAGGTTTTGAGGCTG 3’	51
TRAV9-1, 9-2	5’ TGGTATGTCCAATATCCTGG 3’	5’ GAAACCACTTCTTTCCACTTG 3’	51
TRAV10	5’ CAAGTGGAGCAGAGTCCTC 3’	5’ GAAAGAACTGCACTCTTCAATG 3’	51
TRAV12-1, 12-2, 12-3	5’ CARTGTTCCAGAGGGAGC 3’	5’ AAGATGGAAGGTTTACAGCAC 3’	51
TRAV13-1	5’ CATCCTTCAACCCTGAGTG 3’	5’ TCAGACAGTGCCTCAAACTAC 3’	51
TRAV13-2	5’ CAGCGCCTCAGACTACTTC 3’	5’ CAGTGAAACATCTCTCTCTGC 3’	51
TRAV14	5’ AAGATAACTCAAACCCAACCAG 3’	5’ AGGCTGTGACTCTGGACTG 3’	51
TRAV16	5’ AGTGGAGCTGAAGTGCAAC 3’	5’ GTCCAGTACTCCAGACAACG 3’	51
TRAV17	5’ GGAGAAGAGGATCCTCAGG 3’	5’ CCACCATGAACTGCAGTTAC 3’	51
TRAV18	5’ TCCAGTATCTAAACAAAGAGCC 3’	5’ TGACAGTTCCTTCCACCTG 3’	51
TRAV19	5’ AGGTAACTCAAGCGCAGAC 3’	5’ TGTGACCTTGGACTGTGTG 3’	51
TRAV20	5’ CACAGTCAGCGGTTTAAGAG 3’	5’ TCTGGTATAGGCAAGATCCTG 3’	51
TRAV21	5’ TTCCTGCAGCTCTGAGTG 3’	5’ AACTTGGTTCTCAACTGCAG 3’	51
TRAV22	5’ GTCCTCCAGACCTGATTCTC 3’	5’ CTGACTCTGTGAACAATTTGC 3’	51
TRAV23	5’ TGCTTATGAGAACACTGCG 3’	5’ TGCATTATTGATAGCCATACG 3’	51
TRAV24	5’ CTCAGTCACTGCATGTTCAG 3’	5’ TGCCTTACACTGGTACAGATG 3’	51

TRAV25	5’ GGACTTCACCACGTACTGC 3’	5’ TATAAGCAAAGGCCTGGTG 3’	51
TRAV26-1	5’ GCAAACCTGCCTTGTAATC 3’	5’ CGACAGATTCACTCCCAG 3’	51
TRAV26-2	5’ AGCCAAATTCAATGGAGAG 3’	5’ TTCACTTGCCTTGTAACCAC 3’	51
TRAV27	5’ TCAGTTTCTAAGCATCCAAGAG 3’	5’ CTCACTGTGTACTGCAACTCC 3’	51
TRAV29	5’ GCAAGTTAAGCAAAATTCACC 3’	5’ CTGCTGAAGGTCCTACATTC 3’	51
TRAV30	5’ CAACAACCAGTGCAGAGTC 3’	5’ AGAAGCATGGTGAAGCAC 3’	51
TRAV34	5’ AGAACTGGAGCAGAGTCCTC 3’	5’ ATCTCACCATAAACTGCACG 3’	51
TRAV35	5’ GGTCAACAGCTGAATCAGAG 3’	5’ ACCTGGCTATGGTACAAGC 3’	51
TRAV36	5’ GAAGACAAGGTGGTACAAAGC 3’	5’ ATCTCTGGTTGTCCACGAG 3’	51
TRAV38-1, 38-2	5’ GCACATATGACACCAGTGAG 3’	5’ CAGCAGGCAGATGATTCTC 3’	51
TRAV39	5’ CTGTTCCTGAGCATGCAG 3’	5’ TCAACCACTTCAGACAGACTG 3’	51
TRAV40	5’ GCATCTGTGACTATGAACTGC 3’	5’ GGAGGCGGAAATATTAAAGAC 3’	51
TRAV41	5’ AATGAAGTGGAGCAGAGTCC 3’	5’ TTGTTTATGCTGAGCTCAGG 3’	51
TRAC	5’ GACCAGCTTGACATCACAG 3’	5’ TGTTGCTCTTGAAGTCCATAG 3’	51

TRB gene(s) targeted by primer	External primer sequence	Internal primer sequence	Reference
TRBV2	5’ TCGATGATCAATTCTCAGTTG 3’	5’ TTCACTCTGAAGATCCGGTC 3’	51
TRBV3-1	5’ CAAAATACCTGGTCACACAG 3’	5’ AATCTTCACATCAATTCCCTG 3’	51
TRBV4-1, 4-2, 4-3	5’ TCGCTTCTCACCTGAATG 3’	5’ CCTGCAGCCAGAAGACTC 3’	51
TRBV5-1, 5-3, 5-4	5’ GATTCTCAGGKCKCCAGTTC 3’	5’ CTTGGAGCTGGRSGACTC 3’	51
TRBV5-5, 5-6, 5-7, 5-8	5’ GTACCAACAGGYCCTGGGT 3’	5’ TCTGAGCTGAATGTGAACG 3’	51
TRBV6-1, 6-2, 6-3, 6-5, 6-6, 6-7, 6-8, 6-9	5’ ACTCAGACCCCAAAATTCC 3’	5’ GTGTRCCCAGGATATGAACC 3’	51
TRBV6-4	5’ ACTGGCAAAGGAGAAGTCC 3’	5’ TGGTTATAGTGTCTCCAGAGC 3’	51
TRBV7-1, 7-2, 7-3	5’ TRTGATCCAATTTCAGGTCA 3’	5’ TCYACTCTGAMGWTCCAGCG 3’	51
TRBV7-4, 7-6, 7-7, 7-8, 7-9	5’ CGSWTCTYTGCAGARAGGC 3’	5’ TGRMGATYCAGCGCACA 3’	51
TRBV9	5’ GATCACAGCAACTGGACAG 3’	5’ GTACCAACAGAGCCTGGAC 3’	51
TRBV10-1	5’ CAGAGCCCAAGACACAAG 3’	5’ TGGTATCGACAAGACCTGG 3’	
TRBV10-2	5’ ACCTTGATGTGTCACCAGAC 3’	5’ GGAACACCAGTGACTCTGAG 3’	

TRBV11-1, 11-2, 11-3	5' CGATTTTCTGCAGAGACGC 3'	5' GACTCCACTCTCAAGATCCA 3'	51
TRBV12-3, 12-4, 12-5	5' ARGTGACAGARATGGGACAA 3'	5' CYACTCTGARGATCCAGCC 3'	51
TRBV13	5' AGCGATAAAGGAAGCATCC 3'	5' CATTCTGAACTGAACATGAGC 3'	51
TRBV14	5' CCAACAATCGATTCTTAGCTG 3'	5' ATTCTACTCTGAAGGTGCAGC 3'	51
TRBV15	5' AGTGACCCTGAGTTGTTCTC 3'	5' ATAACTTCCAATCCAGGAGG 3'	51
TRBV16	5' GTCTTTGATGAAACAGGTATGC 3'	5' CTGTAGCCTTGAGATCCAGG 3'	51 (External only)
TRBV17	5' CAGACCCCCAGACACAAG 3'	5' TGTTCACTGGTACCGACAG 3'	51
TRBV18	5' CATAGATGAGTCAGGAATGCC 3'	5' CGATTTTCTGCTGAATTTCC 3'	51
TRBV19	5' AGTTGTGAACAGAATTTGAACC 3'	5' TTCCTCTCACTGTGACATCG 3'	51
TRBV20-1	5' AAGTTTCTCATCAACCATGC 3'	5' ACTCTGACAGTGACCAGTGC 3'	51
TRBV23-1	5' GCGATTCTCATCTCAATGC 3'	5' GCAATCCTGTCCTCAGAAC 3'	51
TRBV24-1	5' CCTACGGTTGATCTATTACTCC 3'	5' GATGGATACAGTGTCTCTCGA 3'	51
TRBV25-1	5' ACTACACCTCATCCACTATTCC 3'	5' CAGAGAAGGGAGATCTTTCC 3'	51
TRBV27, 28	5' TGGTATCGACAAGACCCAG 3'	5' TTCYCCCTGATYCTGGAGTC 3'	51
TRBV29-1	5' TTCTGGTACCGTCAGCAAC 3'	5' TCTGACTGTGAGCAACATGAG 3'	51
TRBV30	5' TCCAGCTGCTCTTCTACTCC 3'	5' AGAATCTCTCAGCCTCCAGAC 3'	51
TRBC	5' TAGAACTGGACTTGACAGCG 3'	5' TTCTGATGGCTCAAACACAG 3'	51

Primers targeting TRAV and TRBV genes are sense. Primers targeting TRAC and TRBC genes are antisense. TRAV, T cell receptor Vα, TRAC, T cell receptor Cα; TRBV, T cell receptor Vβ, TRBC, T cell receptor Cβ.

Supplementary Table 10. CDR3α and CDR3β nucleotide sequences for TCRs listed in Figure 1c, Figure 2c and Supp Table 1.

Figure	CDR3α amino acid sequence	CDR3β amino acid sequence	CDR3α nucleotide sequence	CDR3β nucleotide sequence
Fig. 1c	CALGDSNYQLIW	CAACGAGGAGQNTLYF	tgtgctctgggtgatagcaactatcagttgatctgg	tgtgccgcgtgcggggctgggggggctggtcaaaacaccttgtacttt
	CALGDHSGSWQLIF	CASSLRTGANSDYTF	tgtgctctgggtgatcattctggcagctggcaactcatcttt	tgtgccagcagtctacggacaggggcaaactccgactacaccttc
	CALGDTGNYKYVF	CASSGHNSGNTLYF	tgtgctctgggtgatacaggaaactacaaatacgtcttt	tgtgccagcagtggacataattctggaaatacgctctatttt
	CALGDTGNYKYVF	CASSAVNSGNTLYF	tgtgctctgggtgatacaggaaactacaaatacgtcttt	tgtgccagcagtgcagtgaattctggaaatacgctctatttt
	CAAGITGNTGKLIF	CASSQFRDRGAEQFF	tgtgctgctggtataacaggcaataccggaaaactcatcttt	tgtgccagcagccaattccgggacaggggtgctgagcagttcttc
	CALGPSGGNYKPTF	CASSPTSSNERLFF	tgcgctctgggcccctcaggaggaaactacaaacctacgttt	tgtgccagcagcccgacatcttccaacgaaagattatttttc
	CALNQGGSAKLIF	CASSQDPSNTGQLYF	tgcgctcttaatcaaggagggtctgcgaagctcatcttt	tgtgccagcagccaagacccatcaaacaccgggcagctctacttt
	CALSGANTGKLTF	CASSQDRSNTEVFF	tgcgctctgagtggagctaacactggaaagctcacgttt	tgtgccagcagccaagacaggtcaaacacagaagtcttcttt
	CALRTGGYKVVF	CASSQDSANTEVFF	tgcgctctgcggactggaggctataaagtggtcttt	tgtgccagcagccaagattcagcaaacacagaagtcttcttt
	CALGTGGYKVVF	CASSRDNSNSDYTF	tgcgctctggggactggaggctataaagtggtcttt	tgtgccagcagccgcgacaactcaaactccgactacaccttc
	CASGTGGYKVVF	CASSLEQSNSDYTF	tgcgcttccgggactggaggctataaagtggtcttt	tgtgccagcagcttagaacagtcaaactccgactacaccttc
	CALRNTNTGKLTF	CASSPRHSGNTLYF	tgcgctctgagaaacaccaatacaggcaaattaacctttt	tgtgccagcagcccccgacattctggaaatacgctctatttt
	CALNGGSNAKLTF	CASSPQTGGYEQYF	tgcgctctgaatggaggaagcaatgcaaagctaaccttc	tgtgccagcagcccccagactggggttatgaacagtacttc
	CALTGGSNAKLTF	CASSQGHERLFF	tgcgctctgactggaggaagcaatgcaaagctaaccttc	tgtgccagcagccaagggcacgaaagattatttttc
	CALGSGGNYKPTF	CASSSQNSGNTLYF	tgcgctctgggctcaggaggaaactacaaacctacgttt	tgtgccagcagcagccaaaattctggaaatacgctctatttt
	CALGTGGYKVVF	CASSDFSVEQYF	tgcgctctggggactggaggctataaagtggtcttt	tgtgccagcagtgatttttccgttgaacagtacttc
	CAYYGSSGNKLIF	CASSFRGDNSPLYF	tgcgcttattatgggagcagtggcaacaagctcatcttt	tgtgccagcagtttcaggggtgataattcgcccctctacttt
	CALSDGGQKLVF	CASGDFRAEQFF	tgcgctctgagtgatggtggccagaagctggtttttt	tgtgccagcggtgattttcgggctgagcagttcttc
	CALGGGSALGRLHF	CASSRGGTANTGQLYF	tgtgctctgggtggaggttcagccttagggaggctgcatttt	tgtgctagcagtagaggcggaacagcaaacaccgggcagctctacttt
	CALSVPNNAGAKLTF	CASSLGQGSSYEQYF	tgtgctctgagtgtcccgaataatgcaggtgccaagctcacattc	tgtgccagcagtctcgggcaggggagctcctatgaacagtacttc
	CALRGSGGNYKPTF	CAWSLGTGQDTQYF	tgcgctctgaggggctcaggaggaaactacaaacctacgttt	tgtgcctggagtctagggacagggcaagacacccagtacttt
	CALSGGNYKPTF	CAWSLGQGGNERLFF	tgcgctctgtcaggaggaaactacaaacctacgttt	tgtgcctggagtctaggtcaggggggggaacgaaagattatttttc
Fig. 2c	CIVRVNYGQNFVF	CASSQAGPLHF	tgcatcgtcagagttaactatggtcagaattttgtcttt	tgtgccagcagccaagcgggacccctccacttt
	CIVNPANTGNQFYF	CASRRDYFSYEQYF	tgcatcgtcaatcctgcgaacaccggtaaccagttctatttt	tgtgccagcagaagggactatttctcctacgagcagtacttc
Supp Table 1	CIVRASNTGNQFYF	CSAATGTSGYEQYF	tgcatcgtcagagcttcgaacaccggtaaccagttctatttt	tgcagtgctgctaccgggactagcggctacgagcagtacttc
	CAGKAGNYGQNFVF	CASSTGGQNTGELFF	tgtgcagggaaagctgggaactatggtcagaattttgtcttt	tgtgccagtagtaccggggacagaacaccggggagctgtttttt
	CIVRTSGGSNYKLTF	CAGTYWNEQFF	tgcatcgtcagaacaagtggaggtagcaactataaactgacattt	tgtgccgggacctactggaatgagcagttcttc
	CIVRVAGANSKLTF	CASSYDLAGPYEQYF	tgcatcgtcagagtcgctggagccaatagtaagctgacattt	tgtgccagcagttatgatctagcggggccctacgagcagtacttc
	CAGRNNAGNMLTF	CASNVGIINYGYTF	tgtgctggccgaaataatgcaggcaacatgctcaccttt	tgtgccagcaatgtcgggattattaactatggctacaccttc
