# Supplementary material for: The molecular basis underlying T cell specificity towards citrullinated epitopes presented by HLA-DR4
Source: Nat Commun. 2024 Jul 23;15:6201. doi: 10.1038/s41467-024-50511-w (PMC11266596; doi:10.1038/s41467-024-50511-w)
Supplement: Supplementary file 3 — Reporting Summary [file 41467_2024_50511_MOESM3_ESM.pdf]

nature portfolio

Corresponding author(s): Jamie Rossjohn
NCOMMS-23-42555

Last updated by author(s): 4 July 2024

Reporting Summary

Nature Portfolio wishes to improve the reproducibility of the work that we publish. This form provides structure for consistency and transparency in reporting. For further information on Nature Portfolio policies, see our Editorial Policies and the Editorial Policy Checklist.

Statistics

For all statistical analyses, confirm that the following items are present in the figure legend, table legend, main text, or Methods section.

n/a	Confirmed	
☐	☒	The exact sample size (n) for each experimental group/condition, given as a discrete number and unit of measurement
☐	☒	A statement on whether measurements were taken from distinct samples or whether the same sample was measured repeatedly
☐	☒	The statistical test(s) used AND whether they are one- or two-sided<br>*Only common tests should be described solely by name; describe more complex techniques in the Methods section.*
☒	☐	A description of all covariates tested
☒	☐	A description of any assumptions or corrections, such as tests of normality and adjustment for multiple comparisons
☐	☒	A full description of the statistical parameters including central tendency (e.g. means) or other basic estimates (e.g. regression coefficient) AND variation (e.g. standard deviation) or associated estimates of uncertainty (e.g. confidence intervals)
☐	☒	For null hypothesis testing, the test statistic (e.g. F, t, r) with confidence intervals, effect sizes, degrees of freedom and P value noted<br>*Give P values as exact values whenever suitable.*
☒	☐	For Bayesian analysis, information on the choice of priors and Markov chain Monte Carlo settings
☒	☐	For hierarchical and complex designs, identification of the appropriate level for tests and full reporting of outcomes
☒	☐	Estimates of effect sizes (e.g. Cohen's d, Pearson's r), indicating how they were calculated

Our web collection on statistics for biologists contains articles on many of the points above.

Software and code

Policy information about availability of computer code

Data collection	FACS data: BD FACSDiva 8.0.1 software (BD Immunocytometry Systems). Surface plasmon resonance (SPR) data: Biacore T200 (Cytiva). Crystallography data: macromolecule data collection and processing implemented at the Australian Synchrotron MX2 beamline.
Data analysis	For FACS data analysis: Flowjo v10.9.0 (Three Star); For single cell TCR TRAV/TRBV usage analysis: databank search engines IMGT/V-QUEST (http://www.imgt.org/IMGT_vquest/vquest?livret=0&Option=TcR). For SPR and figure 1b generation: Prism-9 and Prism-10 (Graphpad). For crystallographic procedures: phenix-1.20.1-4487 (https://phenix-online.org), ccp4-8.0 and COOT-0.9.8.91 (https://www.ccp4.ac.uk), XDS automated data processing provided by Australian synchrotron; All structural figures were generated by PyMol v2.5.5.

For manuscripts utilizing custom algorithms or software that are central to the research but not yet described in published literature, software must be made available to editors and reviewers. We strongly encourage code deposition in a community repository (e.g. GitHub). See the Nature Portfolio guidelines for submitting code & software for further information.

Data

Policy information about availability of data

All manuscripts must include a data availability statement. This statement should provide the following information, where applicable:

- Accession codes, unique identifiers, or web links for publicly available datasets
- A description of any restrictions on data availability
- For clinical datasets or third party data, please ensure that the statement adheres to our policy

Data availability. The X-ray crystal structures were deposited in the Worldwide Protein Data Bank (https://www.rcsb.org/) with the following accession codes: A03

TCR-HLA-DR4Vim-64cit59-71, 8TRR (https://www.rcsb.org/structure/unreleased/8TRR); A07 TCR-HLA-DR4Vim-64cit59-71, 8TRQ (https://www.rcsb.org/structure/unreleased/8TRQ); RA2.7 TCR-HLA-DR4a-eno-15cit10-22V20G, 8TRL (https://www.rcsb.org/structure/unreleased/8TRL). All data generated in this study are provided in the Supplementary Information/Source Data File. Source data are provided as a Source Data file. Source data are provided with this paper.

Research involving human participants, their data, or biological material

Policy information about studies with human participants or human data. See also policy information about sex, gender (identity/presentation), and sexual orientation and race, ethnicity and racism.

Reporting on sex and gender	RA donor 2 is male and RA donar 3 is female. 2 healthy donors are both male. Sex and gender based analyses not considered in study design.
Reporting on race, ethnicity, or other socially relevant groupings	RA donor 2 is White, HLA-DRB1*04:01, DRB1*11:01,<br>RA donor 3 is African American, HLA-DRB1*04:01, DRB1*13:03,<br>Both healthy donors were White, HLA-DRB1*04:01, DRB1*04:01.<br>The race and ethnicity is not relevant in this study.
Population characteristics	RA donor 2, male and 66 years old, ACPA+; RA donor 3, female and 93 years old, ACPA-; healthy donor (10195), male, 30 years old and healthy donor (4737), male, 27 years old.
Recruitment	Peripheral blood samples of healthy donors were purchased from AllCells, USA while RA donor PBMC samples were obtained from BioIVT, USA, companies that provide high-quality biological specimens.
Ethics oversight	Human PBMC samples were obtained based on the consent of individual donors for their donation to be utilised in biomedical research. Human experimental work was conducted according to the Australian National Health and Medical Research Council (MHMRC) Code of Practice. Use of PBMC samples for analysis of epitope-specific cells from humans was approved by the Monash University Human Research Ethics Committee (HREC 23019).

Note that full information on the approval of the study protocol must also be provided in the manuscript.

Field-specific reporting

Please select the one below that is the best fit for your research. If you are not sure, read the appropriate sections before making your selection.

[x] Life sciences [ ] Behavioural & social sciences [ ] Ecological, evolutionary & environmental sciences

For a reference copy of the document with all sections, see nature.com/documents/nr-reporting-summary-flat.pdf

Life sciences study design

All studies must disclose on these points even when the disclosure is negative.

Sample size	No sample size calculations were undertaken. Sample size for SPR and T cell stimulation experiments were chosen based on observed reproducibility of experimental outcomes in preliminary experiments: For SPR n≥ 2 independent experiments were performed. These sample sizes have been previously used by our laboratory: Petersen et al Nat Struct Mol Biol. 2020 Jan;27(1):49-61. doi: 10.1038/, s41594-019-0353-4; Beringer et al Nat Immunol. 2015 Nov;16(11):1153-61.doi: 10.1038/ni.3271; For tissue samples (PBMC) isolated from healthy donors and RA donors: No sample size calculations were performed for both human samples (PBMC) and Mice study. The sample size for PBMC and Mice not influence our study because our aim is to identify specific TCR toward peptide MHC, these sample sizes have been previously used by our laboratory: Lim et al., Sci. Immunol. 2021 Apr 16;6(58):eabe0896. doi: 10.1126/sciimmunol.abe0896.
Data exclusions	No data exclusion.
Replication	Each experiment was performed at least twice independently as indicated in the figure legends.
Randomization	Randomisation was not relevant to this study because it was in vitro biochemical based analysis and was not an experimental study that required allocation into groups.
Blinding	Not relevant. This was not a clinical study so no cohort comparison (i.e. different treatment groups) or similar experiment was performed.

Reporting for specific materials, systems and methods

We require information from authors about some types of materials, experimental systems and methods used in many studies. Here, indicate whether each material, system or method listed is relevant to your study. If you are not sure if a list item applies to your research, read the appropriate section before selecting a response.

Materials & experimental systems

n/a	Involved in the study	
	☒	Antibodies
	☒	Eukaryotic cell lines
☒		Palaeontology and archaeology
	☒	Animals and other organisms
☒		Clinical data
☒		Dual use research of concern
☒		Plants

Methods

n/a	Involved in the study	
☒		ChIP-seq
	☒	Flow cytometry
☒		MRI-based neuroimaging

Antibodies

Antibodies used

BUV395 mouse anti-human CD3 (clone UCHT1, Cat # 563546, BD Biosciences); APC Mouse Anti-Human CD69 (Clone FN50, Cat # 555533, BD Biosciences); anti-HLA-DR4 (clone LB3.1); APC-Cy™7 Hamster Anti-Mouse TCR β Chain (clone H57-597, Cat # 560656, BD Biosciences), FITC Rat Anti-Mouse CD45R/B220 (Clone RA3-6B2, BD Biosciences, Cat. no. 553088), FITC Mouse Anti-Mouse NK-1.1 (clone PK136, BD Biosciences, Cat # 553164), FITC Rat anti-Mouse F4/80 (clone BM8, eBioscience, ThermoFisher Scientific, Cat # 11-4801-82), Pacific Blue™ Rat Anti-Mouse CD8a (clone: 53-6.7, BD Biosciences, Cat # 558106), BV711 Hamster Anti-Mouse CD3e (clone: 145-2C11, BD Biosciences, Cat # 563123), BUV395 Rat Anti-Mouse CD4 (clone GK1.5, BD Biosciences, Cat # 563790), BV605 Rat Anti-Mouse CD62L (clone MEL-14, BD Biosciences, Cat # 563252), BV480 Mouse anti-human CD3 (Clone UCHT1, BD Biosciences, Cat # 566105), Alexa Fluor 700 Mouse anti-human CD14 (clone M5E2, BD Biosciences, Cat # 557923), Alexa Fluor 700 Mouse anti-human CD19 (clone: HIB19, BD Biosciences, Cat # 557921), BUV395 mouse anti-human CD4 (clone SK3, BD Biosciences, Cat # 563550), anti-mouse TCRb-APC antibody (clone H57-597, Biolegend Cat. 109212), anti-human CD3-APC antibody (clone UCHT1, Biolegend Cat. 300412),LIVE/DEAD™ Fixable Aqua Dead Cell Stain Kit, for 405 nm excitation LIVE/DEAD™ (Invitrogen, ThermoFisher Scientific, Cat # L34957), Zombie NIR™ Fixable Viability Kit (Biolegend, Cat # 423106), anti-CD16/CD32 mABs (clone 2.4G2, Fc block), anti-human FcR block (Miltenyi Biotech, Cat # 130-059-901), anti-PE microbeads (Miltenyi Biotech, Cat # 130-048-801), anti-APC microbeads (Miltenyi Biotech, Cat # 130-090-855), BD Horizon Fixable viability stain 700 (BD Horizon, Cat # BD564997)

Validation

BUV395 mouse anti-human CD3 (clone UCHT1, cat. no. 563546, BD Biosciences), BV480 Mouse anti-human CD3 (Clone UCHT1, BD Biosciences, Cat # 566105):
PMID: 6788570; Beverley PC, Callard RE. Distinctive functional characteristics of human "T" lymphocytes defined by E rosetting or a monoclonal anti-T cell antibody. Eur J Immunol. 1981; 11(4):329-334. (Clone-specific)
PMID: 6980937; Burns GF, Boyd AW, Beverley PC. Two monoclonal anti-human T lymphocyte antibodies have similar biologic effects and recognize the same cell surface antigen. J Immunol. 1982;129(4):1451-1457.

APC Mouse Anti-Human CD69 (Clone FN50, cat no. 555533, BD Biosciences):
Knapp W. W. Knapp .. et al., ed. Leucocyte typing IV : white cell differentiation antigens. Oxford New York: Oxford University Press; 1989:1-1182.
Schlossman SF. Stuart F. Schlossman .. et al., ed. Leucocyte typing V : white cell differentiation antigens : proceedings of the fifth international workshop and conference held in Boston, USA, 3-7 November, 1993. Oxford: Oxford University Press; 1995.

anti-HLA-DR (clone LB3.1):
PMID: 3005199; doi: 10.1016/0198-8859(86)90023-6; P J Knudsen, J L Strominger. A monoclonal antibody that recognizes the alpha chain of HLA-DR antigens. 1986 Feb;15(2):150-63.
PMID: 3492282; doi: 10.1016/0008-8749(86)90077-8; J C Gorga et al.. Immunochemically purified DR antigens in liposomes stimulate xenogeneic cytolytic T cells in secondary in vitro cultures. 1986 Nov;103(1):160-73.
PMID: 7520896; doi: 10.1016/0198-8859(94)90268-2; X T Fu, R W Karr. HLA-DR alpha chain residues located on the outer loops are involved in nonpolymorphic and polymorphic antibody-binding epitopes. 1994 Apr;39(4):253-60.

APC-Cy™7 Hamster Anti-Mouse TCR β Chain (clone H57-597(RUO), cat. no. 560656, BD Biosciences):
PMID: 7814875 ; Atsuta N, Nishimura H, Nakamura N, Emoto M, Iwatsuki T, Yoshikai Y. Diversity of V gamma gene segments rearranged to the J gamma 4 gene in mice. J Immunol. 1995; 154(2):676-684.
PMID:7907820; Bendelac A, Killeen N, Littman DR, Schwartz RH. A subset of CD4+ thymocytes selected by MHC class I molecules. Science. 1994; 263(5154):1774-1778.

FITC Rat Anti-Mouse CD45R/B220 (Clone RA3-6B2, BD Biosciences, Cat. no. 553088):
PMID:1383316; Allman DM, Ferguson SE, Cancro MP. Peripheral B cell maturation. I. Immature peripheral B cells in adults are heat-stable antigenhi and exhibit unique signaling characteristics. J Immunol. 1992; 149(8):2533-2540.
PMID: 2478453; Asensi V, Kimeno K, Kawamura I, Sakumoto M, Nomoto K. Treatment of autoimmune MRL/lpr mice with anti-B220 monoclonal antibody reduces the level of anti-DNA antibodies and lymphadenopathies. Immunology. 1989; 68(2):204-208. (Clone-specific).

FITC Mouse Anti-Mouse NK-1.1 (clone PK136, BD Biosciences, Cat # 553164):
PMID:9396764; Arase N, Arase H, Park SY, Ohno H, Ra C, Saito T. Association with FcRgamma is essential for activation signal through NKR-P1 (CD161) in natural killer (NK) cells and NK1.1+ T cells. J Exp Med. 1997; 186(12):1957-1963.
PMID: 10229828; Carlyle JR, Martin A, Mehra A, Attisano L, Tsui FW, Zuniga-Pflucker JC. Mouse NKR-P1B, a novel NK1.1 antigen with

inhibitory function. J Immunol. 1999; 162(10):5917-5923. (Clone-specific: Immunoprecipitation)

FITC Rat anti-Mouse F4/80 (clone BM8, eBioscience, ThermoFisher Scientific, Cat # 11-4801-82):
PMID: 36114279; Seo SH,Kim E,Yoon M,Lee SH,Park BH,Choi KY. Metanolic improvement and liver regeneration by inhibiting CXXC5 function for non-alcoholic steatohepatitis treatment. Exp Mol Med. 2022 Sep;54(9):1511-1523.
PMID:35903953; Liu X,Jiang Q,Shen S,Hou Y. Local and systemic inflammation triggers different outcomes of tumor growth related to infiltration of anti-tumor or pro-tumor macrophages. Chin Med J (Engl). 2022 Aug 5;135(15):1821-1828.

Pacific Blue™ Rat Anti-Mouse CD8a (clone: 53-6.7, BD Biosciences, Cat # 558106):
PMID: 2653377; Bierer BE, Sleckman BP, Ratnofsky SE, Burakoff SJ. The biologic roles of CD2, CD4, and CD8 in T-cell activation. Annu Rev Immunol. 1989; 7:579-599.
PMID:8609387; Fujiura Y, Kawaguchi M, Kondo Y, et al. Development of CD8 alpha alpha+ intestinal intraepithelial T cells in beta 2-microglobulin- and/or TAP1-deficient mice. J Immunol. 1996; 156(8):2710-2715.

BV711 Hamster Anti-Mouse CD3e (clone: 145-2C11, BD Biosciences, Cat # 563123):
PMID:8986720; Castro JE, Listman JA, Jacobson BA, et al. Fas modulation of apoptosis during negative selection of thymocytes. Immunity. 1996; 5(6):617-627. (Clone-specific: Activation, Apoptosis)
PMID: 7528772; Isakov N, Wange RL, Burgess WH, Watts JD, Aebersold R, Samelson LE. ZAP-70 binding specificity to T cell receptor tyrosine-based activation motifs: the tandem SH2 domains of ZAP-70 bind distinct tyrosine-based activation motifs with varying affinity. J Exp Med. 1995; 181(1):375-380.

BUV395 Rat Anti-Mouse CD4 (clone GK1.5, BD Biosciences, Cat # 563790):
PMID: 10562325; Bosselut R, Zhang W, Ashe JM, Kopacz JL, Samelson LE, Singer A. Association of the adaptor molecule LAT with CD4 and CD8 coreceptors identifies a new coreceptor function in T cell receptor signal transduction. J Exp Med. 1999; 190(10):1517-1526. (Clone-specific: (Co)-stimulation, Immunoprecipitation).
PMID: 6415170; Dialynas DP, Quan ZS, Wall KA, et al. Characterization of the murine T cell surface molecule, designated L3T4, identified by monoclonal antibody GK1.5: similarity of L3T4 to the human Leu-3/T4 molecule. J Immunol. 1983; 131(5):2445-2451. (Immunogen: Blocking, Depletion, Flow cytometry)

BV605 Rat Anti-Mouse CD62L (clone MEL-14, BD Biosciences, Cat # 563252):
PMID: 9647212; Cerwenka A, Carter LL, Reome JB, Swain SL, Dutton RW. In vivo persistence of CD8 polarized T cell subsets producing type 1 or type 2 cytokines. J Immunol. 1998; 161(1):97-105. (Biology: Blocking, Flow cytometry, Immunoaffinity chromatography, Immunoprecipitation).
PMID: 6866086; Gallatin WM, Weissman IL, Butcher EC. A cell-surface molecule involved in organ-specific homing of lymphocytes. Nature. 1983; 304(5921):30-34. (Immunogen).

Alexa Fluor 700 Mouse anti-human CD14 (clone M5E2, BD Biosciences, Cat # 557923):
Knapp W. W. Knapp .. et al., ed. Leucocyte typing IV : white cell differentiation antigens. Oxford New York: Oxford University Press; 1989:1-1182.
PMID: 1698311; Wright SD, Ramos RA, Tobias PS, Ulevitch RJ, Mathison JC. CD14, a receptor for complexes of lipopolysaccharide (LPS) and LPS binding protein. Science. 1990; 249(4975):1431-1433.

Alexa Fluor 700 Mouse anti-human CD19 (clone: HIB19, BD Biosciences, Cat # 557921):
PMID: 7690791; Bradbury LE, Goldmacher VS, Tedder TF. The CD19 signal transduction complex of B lymphocytes. Deletion of the CD19 cytoplasmic domain alters signal transduction but not complex formation with TAPA-1 and Leu 13. J Immunol. 1993; 151 (6):2915-2927.
PMID: 2665173; Favaloro EJ, Moraitis N, Koutts J, Exner T, Bradstock KF. Endothelial cells and normal circulating haemopoietic cells share a number of surface antigens. Thromb Haemost. 1989; 61(2):217-224.

BUV395 mouse anti-human CD4 (clone SK3, BD Biosciences, Cat # 563550):
PMID: 6454755; Engleman EG, Benike CJ, Glickman E, Evans RL. Antibodies to membrane structures that distinguish suppressor/ cytotoxic and helper T lymphocyte subpopulations block the mixed leukocyte reaction in man. J Exp Med. 1981; 154(1):193-198.
PMID: 6787593; Evans RL, Wall DW, Platsoucas CD, et al. Thymus-dependent membrane antigens in man: inhibition of cell-mediated lympholysis by monoclonal antibodies to TH2 antigen. Proc Natl Acad Sci U S A. 1981; 78(1):544-548. (Immunogen: Flow cytometry, Inhibition).

anti-mouse TCRb-APC antibody (clone H57-597, Biolegend Cat. 109212):
PMID: 33562706; Grigsby SM, et al. Elucidating the Importance of DOT1L Recruitment in MLL-AF9 Leukemia and Hematopoiesis. Cancers (Basel). 2021 Feb 5;13(4):642.
PMID: 34289356; Chappaz S, et al. Homeostatic apoptosis prevents competition-induced atrophy in follicular B cells. Cell Rep. 2021 Jul 20; 36 (3): 109430.

anti-human CD3-APC antibody (clone UCHT1, Biolegend Cat. 300412):
PMID:32719138; Crawford MP, et al. CD4 T cell-intrinsic role for the T helper 17 signature cytokine IL-17: Effector resistance to immune suppression. PNAS. 2020 Aug 11;117(32):19408-19414.
PMID: 29331015; Toepfner N et al. Detection of human disease conditions by single-cell morpho-rheological phenotyping of blood. Elife. 2018 Jan 13; 7:e29213.

LIVE/DEAD™ Fixable Aqua Dead Cell Stain Kit, for 405 nm excitation LIVE/DEAD™ (Invitrogen, ThermoFisher Scientific, Cat # L34957)
PMID:19168629; Idoyaga J, et al. Antibody to Langerin/CD207 localizes large numbers of CD8alpha+ dendritic cells to the marginal zone of mouse spleen. PNAS. 2009 Feb 3:106(5):1524-9.

PMID:19380804; George Makedonas et.al. Rapid up-regulation and granule-independent transport of perforin to the immunological synapse define a novel mechanism of antigen-specific CD8+ T cell cytotoxic activity. J Immunol. 2009 May 1;182(9):5560-9.

Zombie NIR™ Fixable Viability Kit (Biolegend, Cat # 423106):
PMID: 26982733; Headley M, et al. Visualization of immediate immune responses to pioneer metastatic cells in the lung. Nature. 2016 Mar 24;531(7595):513-7.
PMID: 32760720; Wang L, et al. SLIT2 Overexpression in Periodontitis Intensifies Inflammation and Alveolar Bone Loss, Possibly via the Activation of MAPK Pathway. Front Cell Dev Biol. 2020 Jul 14; 8:593.

anti-CD16/CD32 mABs (clone 2.4G2, Fc block):
PMID: 25038257; Xinmei.Z et al. Scavenger receptor function of mouse Fcγ receptor III contributes to progression of atherosclerosis in apolipoprotein E hyperlipidemic mice. J Immunol. 2014 Sep 1;193(5):2483-95.
PMID: 27967303; Lydia Kwast et al. Immune responses induced by diclofenac or carbamazepine in an oral exposure model using TNP-Ficoll as reporter antigen. J Immunotoxicol. 2016 Nov; 13(6):918-926.

anti-human FcR block (Miltenyi Biotech, Cat # 130-059-901), :
PMID: 20440073; La Gruta NL et al. Primary CTL response magnitude in mice is determined by the extent of naive T cell recruitment and subsequent clonal expansion. J Clin Invest. 2010: 120, 1885-1894.
PMID: 17707129; Moon JJ, et al. Naive CD4(+) T cell frequency varies for different epitopes and predicts repertoire diversity and response magnitude. Immunity. 2007: 27, 203-213.

anti-PE microbeads (Miltenyi Biotech, Cat # 130-048-801), anti-APC microbeads (Miltenyi Biotech, Cat # 130-090-855):
PMID: 15162426; Barnes, E. et al. Ultra-sensitive class I tetramer analysis reveals previously undetectable populations of antiviral CD8 + T cells. Eur J Immunol. 2004 Jun;34(6): 1570-7.
PMID: 15194806; Lucas, M. et al. Ex vivo phenotype and frequency of influenza virus-specific CD4 memory T cells. J Virol. 2004 Jul; 78 (13): 7284-7.

BBD Horizon Fixable viability stain 700 (BD Horizon, Cat # BD564997):
PMID: 19135024; Barny Abrams et al. 3-Carboxy-6-chloro-7-hydroxycoumarin: ahighly fluorescent, water-soluble violet-excitable dye for cell analysis. Anal Biochem. 2009 March 15; 386(2):262-9.
PMID: 18178815; Yvonne Burmeister et al. ICOS controls the pool size of effector-memory and regulatory T cells. J Immunol.2008 Jan15;180 (2):774-82

Eukaryotic cell lines

Policy information about cell lines and Sex and Gender in Research

Cell line source(s)

HEK293T(CRL-3216) and HEK293S GnTI- (CRL-3022) cells were from ATCC. BLCL lines were sourced from the The International Histocompatibility Working Group (IHWG) Cell and DNA Bank, SKW3 line (ACC 53) was sourced from German Collection of Microorganisms and Cell Cultures (DSMZ).

Authentication

HLA-DR expression was confirmed on 9031 BLCL cells by staining with anti-DR monoclonal antibody and FACS analyses. Absence of CD3 expression was confirmed on SKW3 parental cells by staining with anti-CD3 monoclonal antibody and subsequent FACS analyses. T cell receptor (TCR) transduction of SKW3 cells was confirmed by IRES driven expression of GFP- and RFP-reporter proteins, (TCR a-chain and TCR b-chain, respectively) and were antibody stained to confirm CD3 (indicating TCR surface expression). HEK293T and HEK293S GnTi- cells from ATCC were not authenticated.

Mycoplasma contamination

Absence of mycoplasma contamination in cell lines was confirmed via PCR.

Commonly misidentified lines
(See ICLAC register)

No commonly misidentified cell lines were used in the study.

Animals and other research organisms

Policy information about studies involving animals; ARRIVE guidelines recommended for reporting animal research, and Sex and Gender in Research

Laboratory animals

Transgenic mice expressing HLA-DR4 (HLA-DRA1*01:01/DRB1*04:01) on a mouse MHC class II knockout background were purchased from Taconic Biosciences (model 4149). Naïve 13- to 20-week-old mice were used in this study.

Wild animals

no wild animals involved in this study

Reporting on sex

2 female and 4 male mouse were used in this study. Sex and gender based analyses not considered in study design.

Field-collected samples

no field-collected samples involved in this study

Ethics oversight

HLA-DR4 mice were housed in the animal facility at Monash University, and all animal experimentation were approved and conducted under guidelines set by the Monash University Animal Ethics Committee.

Note that full information on the approval of the study protocol must also be provided in the manuscript.

Flow Cytometry

Plots

Confirm that:

- [x] The axis labels state the marker and fluorochrome used (e.g. CD4-FITC).
- [x] The axis scales are clearly visible. Include numbers along axes only for bottom left plot of group (a 'group' is an analysis of identical markers).
- [x] All plots are contour plots with outliers or pseudocolor plots.
- [x] A numerical value for number of cells or percentage (with statistics) is provided.

Methodology

Sample preparation	HLA-DR4-Vim64cit and HLA-DR4-a-enol15cit restricted CD4+ T cells sorted from immunized mice : single-cell suspensions of draining lymph nodes and spleen (inguinal and popliteal) from day 8-immunized HLA-DR4 mice were stained with phycoerythrin(PE)- or allophycocyanin (APC)-labelled DR4 tetramers for 1 h at room temperature, washed, and labelled with anti-PE- or anti-APC- conjugated magnetic microbeads, and tetramer-bound cells were enriched over a magnetic LS column (Miltenyi Biotec). Enriched cells were then stained with a cocktail of conjugated antibodies to identify epitope-specific cells from immune mouse CD4+ T cell populations (B220, F4/80, NK1.1, TCRb, CD8, CD4, CD3 and CD62L).<br><br>HLA-DR4-Vim64cit and HLA-DR4-a-enol15cit restricted CD4+ T cells sorted from human PBMC: cryopreserved PBMC were thawed and rested overnight at 37°C, 5% CO2. Cells were counted and 22-27 million (RA donor 2), 12 million (RA donor 3) or 50 million (healthy donor) PBMC were treated with 50 nM dasatinib for 30 min at 37 °C, then stained with PE- or APC-labelled tetramers (at 10 μg/ml final concentration) for 1 h at room temperature. Cells were then washed and labelled with anti-PE or anti-APC conjugated magnetic microbeads, and tetramer-bound cells were enriched over a magnetic LS column (Miltenyi Biotec). Enriched cells were then stained with a cocktail of conjugated antibodies to identify epitope-specific cells from naïve CD4+ T cell populations (CD14, CD19, CD3, CD4, and FVS700 or Live/Dead Fixable Near-IR viability stain).<br><br>For T cell stimulation assay and In vitro TCR expression : Cells were harvested by centrifugation and resuspended/washed with Phosphate buffered saline (PBS). PBS containing 2% fetal bovine serum was used for antibody and or HLA-DR4 tetramer staining.
Instrument	FACSAria III cell sorter, BD LSRFortessa™ X-20
Software	FlowJo v10
Cell population abundance	For single-cell sorting samples a 'post-sort' flow cytometry analysis was not possible.
Gating strategy	HLA-DR4-Vim64cit and HLA-DR4-a-enol15cit restricted CD4+ T cells sorted from immunized mice (draining lymph nodes and spleen) were gated as follows: single cells (FSC-A/FSC-H); lymphocytes (FSC-A/SSC-A); TCR positive, dump negative cells (TCRb-APC-Cy7 high, B220-F4/80-NK1.1-FITC low); live cells (Aqua Blue viability stain, BV525-A low); activated CD3+ cells (CD3-BV711 high/CD62L-BV605 low); CD4+ T cells (CD4-BUV395 high/CD8-Pacific Blue low).<br><br>HLA-DR4-Vim64cit and HLA-DR4-a-enol15cit restricted CD4+ T cells sorted from human PBMC were gated as follows: single cells (FSC-A/FSC-H); live cells (FVS700 or Live/Dead Fixable Near-IR); lymphocytes (FSC-A/SSC-A); CD3 positive, dump negative cells (CD3-BV480 high, CD14-CD19-AF700 low); CD4+ T cells (CD4-BUV395 high, CD3-BV480 high).<br><br>T cell stimulation assay - RA2.7 TCR (GFP/RFP) transduced SKW3 cells were gated as follows: cells (FCS-A/SSC-H); single cells (FSC-A/FSC-H); live cells (Zombie NIR viability stain, RB780-A low); TCR:GFP expression (B530-A high), RFP expression (YG586 high); human CD3 (CD3-BUV-395) versus human CD69 (APC-CD69) was analyzed to observed the CD3 down regulation and CD69 up-regulation upon T cell activation.<br><br>In vitro TCR expression using HEK293T cells were gated as follows: Cells (FCS-A/SSC-H); single cells (FSC-A/FSC-H); live cells (Aqua Blue viability stain, BV525-A low); GFP high (eGFP B530); Tetramer (PE YG585) versus mouse TCR (TCR-APC or TCR-APC-Cy7) or human CD3 (CD3-BUV395).

- [x] Tick this box to confirm that a figure exemplifying the gating strategy is provided in the Supplementary Information.
